# Zinc Supplementation Protects against Cadmium Accumulation and Cytotoxicity in Madin-Darby Bovine Kidney Cells

**DOI:** 10.1371/journal.pone.0103427

**Published:** 2014-08-08

**Authors:** Ding Zhang, Jingying Liu, Jianfeng Gao, Muhammad Shahzad, Zhaoqing Han, Zhi Wang, Jiakui Li, Hong Sjölinder

**Affiliations:** 1 College of Veterinary Medicine, Huazhong Agricultural University, Wuhan, PR China; 2 Department of Molecular Biosciences, The Wenner-Gren Institute, Stockholm University, Stockholm, Sweden; Instituto Nacional de Cardiologia, Mexico

## Abstract

Cadmium ions (Cd^2+^) have been reported to accumulate in bovine tissues, although Cd^2+^ cytotoxicity has not been investigated thoroughly in this species. Zinc ions (Zn^2+^) have been shown to antagonize the toxic effects of heavy metals such as Cd^2+^ in some systems. The present study investigated Cd^2+^ cytotoxicity in Madin-Darby bovine kidney (MDBK) epithelial cells, and explored whether this was modified by Zn^2+^. Exposure to Cd^2+^ led to a dose- and time-dependent increase in apoptotic cell death, with increased intracellular levels of reactive oxygen species and mitochondrial damage. Zn^2+^ supplementation alleviated Cd^2+^-induced cytotoxicity and this protective effect was more obvious when cells were exposed to a lower concentration of Cd^2+^ (10 μM), as compared to 50 μM Cd^2+^. This indicated that high levels of Cd^2+^ accumulation might induce irreversible damage in bovine kidney cells. Metallothioneins (MTs) are metal-binding proteins that play an essential role in heavy metal ion detoxification. We found that co-exposure to Zn^2+^ and Cd^2+^ synergistically enhanced RNA and protein expression of MT-1, MT-2, and the metal-regulatory transcription factor 1 in MDBK cells. Notably, addition of Zn^2+^ reduced the amounts of cytosolic Cd^2+^ detected following MDBK exposure to 10 μM Cd^2+^. These findings revealed a protective role of Zn^2+^ in counteracting Cd^2+^ uptake and toxicity in MDBK cells, indicating that this approach may provide a means to protect livestock from excessive Cd^2+^ accumulation.

## Introduction

Cadmium (Cd) is a heavy metal that is extensively used in the manufacture of alloys, pigments, electroplates, and batteries. The toxic effects of free cadmium ions (Cd^2+^) have been studied intensively in humans, and effects on a wide range of organs have been reported, including the liver, bones, kidneys, and the reproductive, neurological, and immunological system [1], [2]. Acute Cd^2+^ toxicity in the respiratory and digestive systems causes severe chemical pneumonitis and bloody diarrhea, respectively [3]. However, the kidney and skeleton are most affected by chronic Cd^2+^ toxicity. With chronic exposure, around 50% of the absorbed Cd^2+^ accumulates in the kidneys, and syndromes associated with Cd^2+^-induced renal damage include impaired vitamin metabolism, proteinuria, and loss of bone calcium [4]. Even though Cd exposure has traditionally been thought to occur in industrializing developing counties because of environmental pollution, it is causing growing concern worldwide because Cd^2+^ can accumulate over time in animals and plants used in human food products [5]. For example, Cd^2+^ accumulation to levels high enough to cause toxic effects in humans was reported in a polish study of cattle in 1999 [6].

Following its absorption into cells, Cd^2+^ complexes with members of the metallothionein (MT) family of conserved low-molecular-weight cysteine- and metal-rich proteins. In mammals, MTs exist mainly in the cytoplasm, but can also be detected in lysosomes, mitochondria, and nuclei. Four MT isoforms, designated MT-1 to MT-4, have been identified. MT-1 and MT-2 are the predominant isoforms and are expressed in most tissues, whereas MT-3 and MT-4 are constitutively expressed in the central nervous system and the stratified squamous epithelium, respectively [7]. A wide range of metals rapidly induce MT-1 and MT-2 transcription via metal-regulatory transcription factor 1 (MTF-1) binding to the metal-responsive elements (MREs) within their promoter regions [8]. In addition, cellular stressors, hormones, reactive oxygen species (ROS), and cytokines can also affect MT gene transcription [9]. MTs play an essential role in the homeostasis of essential metal ions, in addition to the sequestration and detoxification of Cd^2+^ and other heavy metals. Furthermore, MTs are efficient scavengers of free radicals generated during oxidative stress [10]. Free Cd^2+^ levels can increase owing to either excessive exposure to Cd^2+^ or MT deficiency, and this can lead to a wide variety of cytotoxic effects.

In humans, Cd^2+^ induces apoptosis via both caspase-dependent and -independent pathways [11]. Caspases are aspartate-specific cysteine proteases that trigger proteolytic cascades and induce amplification of intracellular apoptotic signals. In human kidney proximal tubule cells, Cd^2+^ was found to induce activation of caspase-9 and caspase-3, probably via the release of cytochrome *c* from damaged mitochondria [12]. Caspase-independent apoptosis can occur by Cd^2+^-mediated effects on the tumor suppressor protein (p53), because Cd^2+^ can replace Zn^2+^ within p53 and thereby compromise p53-mediated DNA damage repair or cell cycle arrest [11]. Cd^2+^ can also activate the Ca^2+^-dependent protease, calpain, which plays an essential role in Cd^2+^-induced caspase-independent apoptosis at early time points in rat kidney proximal tubule cells [12]. Cd^2+^-induced apoptosis is associated with ROS accumulation, which can induce mitochondrial, DNA, and protein damage [13].

Zinc (Zn) is an essential trace element that plays a pivotal role in the structural stability of Zn^2+^-dependent proteins and in antagonizing the toxic effects of heavy metals such as Cd^2+^ [14], [15], although exposure to Zn^2+^ can also accelerate apoptosis [16]. Zn^2+^ supplementation counteracted acute Cd^2+^-induced nephrotoxicity in a mouse model [17] and the amounts of Cd^2+^ identified in cattle were inversely proportional to the levels of Zn^2+^ [6]. However, little is known about Cd^2+^-mediated toxicity in cows, or the protective effects of Zn^2+^ supplementation in this animal. This issue has direct economic relevance to food production and important indirect consequences for global public health. The objectives of this study were therefore to investigate Cd^2+^ toxicity in bovine kidney cells and to explore whether Zn^2+^ supplementation prevented Cd^2+^ absorption and/or cytotoxicity.

## Materials and Methods

### Cell culture and treatment

The Madin-Darby bovine kidney epithelial cell line (MDBK, obtained from Boster, Wuhan, China) was maintained in RPMI 1640 medium supplemented with 10% heat-inactivated fetal bovine serum (FBS) at 37°C in a humidified atmosphere with 5% CO_2_. Cells were seeded into a 6-well cell culture plate and grown to approximately 80% confluence before exposure to CdCl_2_ alone, or in combination with ZnCl_2_, for the indicated time periods. Control cells were treated with growth medium (RPMI with 10% FBS).

### Measurement of cell damage

The number of cells undergoing apoptosis and necrosis was determined by flow cytometry after staining with fluorescein isothiocyanate (FITC)-conjugated annexin V antibody and propidium iodide (PI) according to the manufacturer's instructions. Data were acquired by FACS Calibur (BD Biosciences, San Diego, CA, USA) and analyzed using FlowJo 7.6.4 software (Tree Star Inc., Ashland, OR, USA).

### RNA isolation and real-time polymerase chain reaction (PCR)

Total RNA was extracted from cells using the TRI Reagent (Invitrogen, USA) and reverse transcribed using a cDNA Synthesis Kit (DingGuo, TER016-2, Wuhan, China). Real-time PCR was performed on a 7500 real-time PCR system (Applied Biosystems, USA) using SYBR-Green. mt-1, *mt-2*, *mtf-1*, and glyceraldehyde 3-phosphate dehydrogenase (gapdh) were amplified using the primers shown in Table S1. Each assay was carried out in a 20-μl reaction mixture containing 50 ng of cDNA, 0.2 μM of each primer, 10 μl of reaction mix, and 0.5 μl of SYBR-Green dye (TaKaRa, Dalian, China). The thermal cycling conditions included an initial denaturation step of 94°C for 4 min, followed by 40 amplification cycles of 92°C for 1 min, 60°C for 1 min, and 72°C for 1 min. Threshold cycle (Ct) values of the target genes were normalized to that of the reference gene (GAPDH) and expressed as fold changes, compared to those associated with the control sample.

### Immunofluorescence staining

Cells were fixed with 4% neutral formaldehyde for 20 min and permeabilized with 0.1% Triton X-100 for 20 min; unspecific binding sites were blocked with 10% bovine serum albumin (BSA) for 30 min before immunofluorescence staining. Cells were incubated with a mouse anti-MT antibody (ab12228, 1∶150, Abcam, Hong Kong, China) that reacts with both MT1 and MT2 or a rabbit anti-MTF-1 antibody (1∶200, EIAab, Wuhan, China) overnight at 4°C, followed by incubation with FITC- or Texas red-conjugated secondary antibodies (1∶100, Proteintech Group Inc., Wuhan, China) for 30 min. Controls for unspecific binding of antibodies were included in each staining. Images of cells were captured using a confocal fluorescence microscope (NOL-LSM710, Carl Zeiss Jena, Germany) and analyzed using ZEN 2009 light edition software.

### Western blot analysis

Cells were lysed in 50 μl of lysis buffer (25 mM Tris, 150 mM NaCl, 1% NP-40, and 0.1 mM SDS). The protein concentration was determined and equal amounts of protein were subjected to 12% sodium dodecyl sulfate-polyacrylamide gel electrophoresis. Proteins were transferred onto polyvinylidene fluoride membranes, and these were incubated with a mouse anti-MT antibody (1∶400, Abcam, Hong Kong, China) or a rabbit anti-MTF-1 antibody (1∶500, EIAab, Wuhan, China). Biotinylated goat anti-mouse or goat anti-rabbit IgG (1∶8000, Boster Company, Wuhan, China) was used as the secondary antibody. Signals were detected using an enhanced chemiluminescence kit (Beyotime, Shanghai, China).

### Measurement of intracellular ROS

Levels of intracellular ROS were detected using a method reported previously [18]. Briefly, treated cells were incubated with 10 μM 2′-7′-dichlorodihydrofluorescein diacetate (DCFH-DA) probes suspended in serum-free medium at 37°C for 30 min. After washing with phosphate-buffered saline (PBS) three times, ROS were monitored by following the intracellular conversion of DCFH-DA into the fluorescent product, dichlorofluorescein (DCF), using either fluorescence microscopy or flow cytometry.

### Measurement of the mitochondrial membrane potential

The mitochondrial membrane potential (MMP) was measured using 5,5′,6,6′-tetrachloro-1,1′,3,3′-tetraethylbenzimidazolcarbocyanine iodide (JC-1), a cationic lipophilic fluorescent dye that can selectively enter mitochondria and reflect MMP through reversible color changes. JC-1 forms complexes and produced red fluorescence in healthy cells exhibiting high MMP; in apoptotic cells with low MMP, JC-1 remains in a monomeric form and emits green fluorescence. In brief, treated cells were incubated with 5 μg/ml of JC-1 for 20 min at 37°C. After intensive washing, the relative amounts of mitochondrial red and green fluorescence were determined by either flow cytometry or fluorescence microscope.

### Quantification of intracellular Cd^2+^ and Zn^2+^

Levels of intracellular Cd^2+^ and Zn^2+^ were quantified by quadrupole inductively coupled plasma mass spectrometry (ICP-MS). Cells were intensively washed with PBS, quantified, and transferred to acid-washed high-density polyethylene bottles. Cells were digested with HNO_3_/HCl (1∶3) at room temperature for 1 day and then completely evaporated by heating. The sediments were re-dissolved with 0.1% HNO_3_ to a volume of 6 ml. Metals were measured using the following operating conditions: 0.8 L/min gas box nebulizer flow, 0.6 L/min gas box auxiliary flow, 10 s acquisition time, three replicates, and 2400 V radio frequency power.

### Statistical analysis

All experiments were repeated at least three times. Statistical analysis was performed using the Statistical Package for the Social Sciences (SPSS) 17.0 software (SPSS Inc., IL, USA). All data are expressed as the mean ± standard deviation (SD) of one representative experiment, performed in quadruplicate. Paired Student's *t*-tests were used to analyze the effect of Cd^2+^- or Zn^2+^-exposure, where *P*<0.05 was considered statistically significant.

## Results

### Effects of Zn^2+^ and Cd^2+^ on apoptosis in MDBK cells

**Figure 1:**
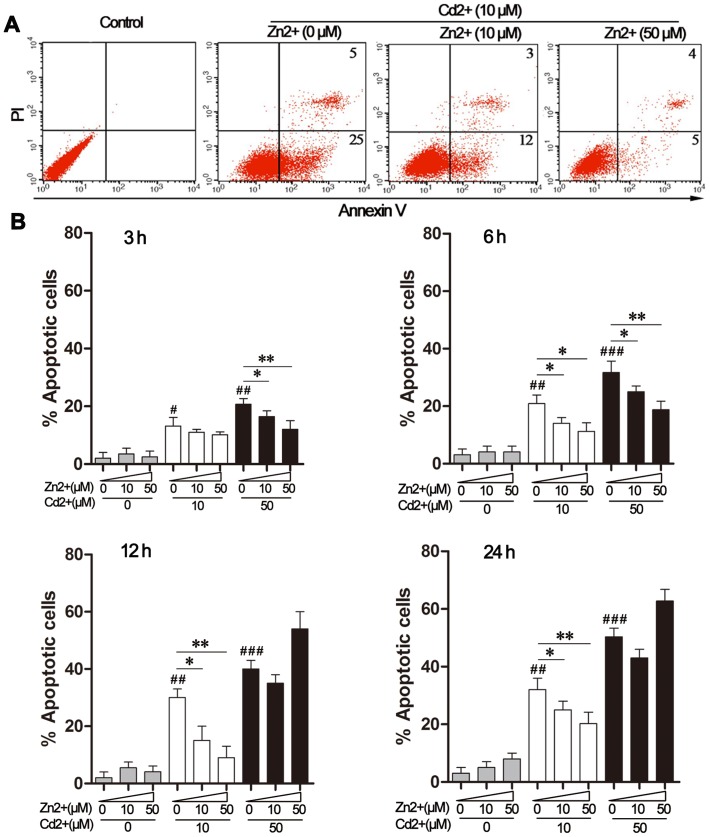
Effects of Zn^2+^ and Cd^2+^ on MDBK apoptosis. (A) MDBK cells were treated with 10 μM CdCl_2_, alone or in combination with ZnCl_2_ (0, 10, or 50 μM), for 12 h. Apoptotic cell death was quantified by flow cytometry following double staining with propidium iodide (PI) and a fluorescein isothiocyanate (FITC)-conjugated annexin V antibody. Control cells were treated with medium. (B) MDBK cells were treated with CdCl_2_ alone (0, 10, or 50 μM) or in combination with ZnCl_2_ (0, 10, or 50 μM) for the indicated time periods. The percentage of PI- or annexin V-positive apoptotic cells was quantified by flow cytometry. The data were expressed as the mean ± SD (n = 4). #*P*<0.05, ##*P*<0.01, and ###*P*<0.001 for the comparison with the medium-treated control group; *P<0.05 and **P<0.01 compared to cells exposed to Cd^2+^ only (10 or 50 μM).

Cd^2+^ and Zn^2+^ have both been shown to accelerate apoptosis in dose- and species-dependent manners [16], [19], [20] and we therefore determined the concentrations of these ions that induced toxic effects on MDBK cells. Cells were treated with 10–100 μM of CdCl_2_ or 10–200 μM of ZnCl_2_ for 12 h and cell survival was analyzed by flow cytometry with staining of annexin V and PI, to detect early and late apoptosis. We observed that 10 μM Cd^2+^ induced mild apoptotic cell damage (Fig. 1A), whereas 50 μM Cd^2+^ caused severe cell death (Fig. 1B). Nearly all cells treated with 100 μM CdCl_2_ underwent apoptosis (data not shown). We found that Cd^2+^-triggered cytotoxicity was both time- and dose-dependent. Compared to these cytotoxic effects of Cd^2+^, addition of 10 or 50 μM Zn^2+^ did not induce apoptosis (Fig. 1B). Based on these findings, 10 μM or 50 μM Cd^2+^ was used subsequently to induce mild or severe toxicity, respectively, for investigation of the possible protective role of Zn^2+^ supplementation (10 or 50 μM).

When cells were exposed to 10 μM CdCl_2_, supplementation with 10 μM or 50 μM Zn^2+^ produced comparable improvements in cell survival up to 24 h after treatment (Fig. 1B). In contrast, Zn^2+^-mediated protection could only be detected at earlier time points (3 h and 6 h) when 50 μM CdCl_2_ was present. After 12-h and 24-h exposure to 50 μM Cd^2+^, addition of 50 μM Zn^2+^ even intensified Cd^2+^-induced cytotoxicity (Fig. 1B). These results suggested that Zn^2+^ prevented mild Cd^2+^-induced cell damage.

### Effect of Zn^2+^ on Cd^2+^-induced ROS accumulation in MDBK cells

**Figure 2:**
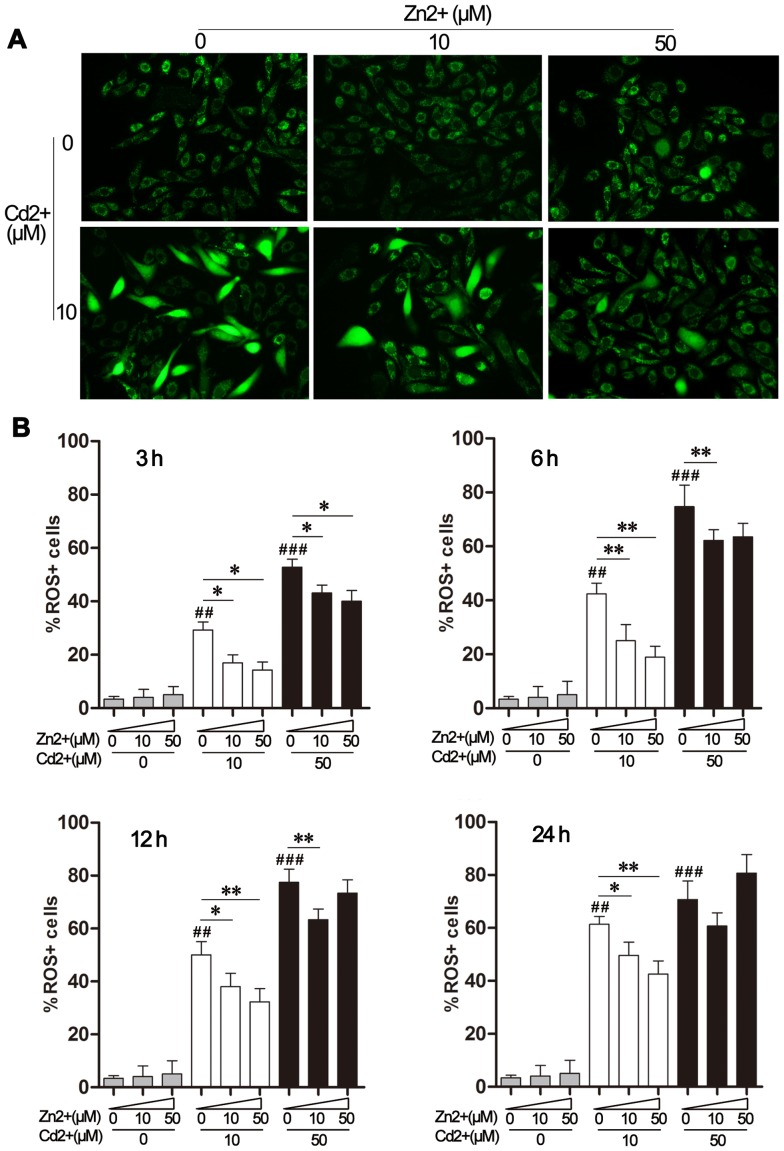
Effect of Zn^2+^ on Cd^2+^-induced accumulation of reactive oxygen species (ROS) in MDBK cells. (A) MDBK cells were treated with 10 μM CdCl_2_, alone or in combination with ZnCl_2_ (0, 10, or 50 μM), for 6 h. Intracellular ROS accumulation was assessed by DCFH-DA staining and imaged using a fluorescence microscope at ×200 magnification. (B) Cells were treated with Zn^2+^ and/or Cd^2+^ as indicated. Intracellular ROS was determined by flow cytometry following staining with DCFH-DA and the percentage of ROS-positive cells was quantified. The data represent the mean ± SD (n = 4). ##*P*<0.01, and ###*P*<0.001, compared to the medium-treated control group; *P<0.05 and **P<0.01 compared to the cells exposed to Cd^2+^ alone (10 or 50 μM).

ROS accumulation plays both direct and indirect roles in inducing apoptosis [21]. We therefore analyzed the levels of ROS, as detected by DCFH-DA conversion to DCF, in MDBK cells exposed to Cd^2+^ and/or Zn^2+^ at the indicated concentrations. As shown in Fig. 2A, strong DCF fluorescence intensity was observed in cells treated with 10 μM Cd^2+^ for 6 h, whereas the addition of Zn^2+^ alone did not cause an obvious increase in ROS levels. Co-treatment with Cd^2+^ and Zn^2+^ led to a lower ROS level in the MDBK cells, as compared to treatment with Cd^2+^ alone.

A detailed time course analysis of the protective effects of Zn^2+^ on Cd^2+^-induced ROS accumulation was performed. MDBK cells were exposed to the indicated concentrations of Cd^2+^ and Zn^2+^ for 3–24 h, and the percentage of ROS positive cells was quantified by flow cytometry analysis. As shown in Fig. 2B, ROS accumulation during MDBK exposure to 10 μM Cd^2+^ was time-dependent, and ROS accumulation peaked at 24 h. However, exposure to 50 μM Cd^2+^ caused a sharp rise in the ROS level at 6 h. Prolonged exposure did not result in an increased number of ROS-positive cells, which might reflect the Cd^2+^-induced cell death observed in Fig. 1B. Addition of either 10 μM or 50 μM Zn^2+^ was associated with reduced ROS levels in cells exposed to 10 μM Cd^2+^ at all of the time points tested. When cells were exposed to 50 μM Cd^2+^, the presence of Zn^2+^ reduced ROS accumulation slightly at earlier time points (up to 12 h), but not at 24 h (Fig. 2B). Consistent with the apoptosis data presented in Fig. 1B, the presence of 50 μM Zn^2+^ even synergized Cd^2+^-induced ROS in MDBK cells; this was in agreement with previous observations in other species, where relatively high concentrations of Zn^2+^ led to elevated levels of ROS and cytotoxicity [22].

### Effect of Zn^2+^ on Cd^2+^-induced mitochondrial depolarization

**Figure 3:**
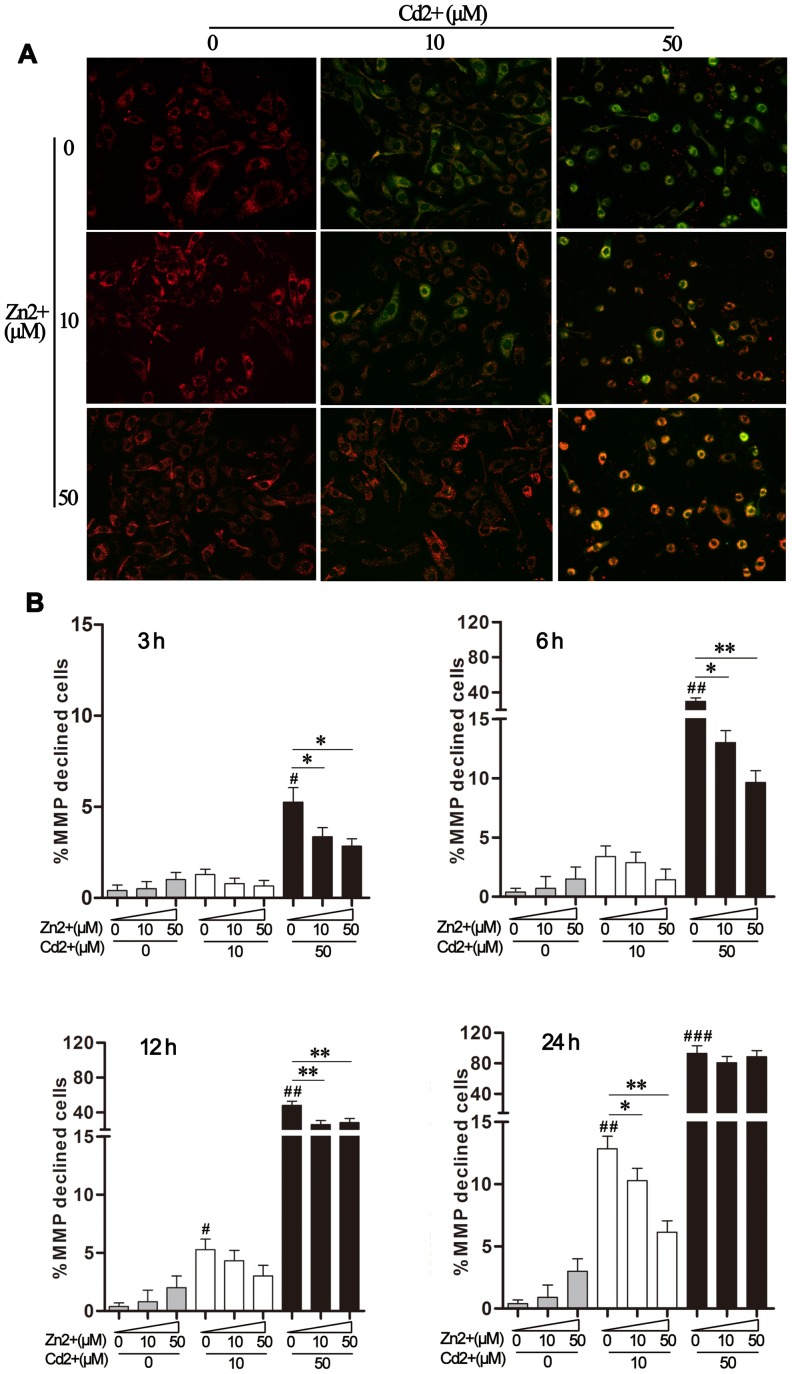
Effect of Zn^2+^ on Cd^2+^-triggered mitochondrial depolarization. (A) MDBK cells were treated with Cd^2+^ alone (0, 10, 50 μM), or in combination with Zn^2+^ (0, 10, 50 μM), for 6 h. Mitochondrial membrane polarization (MMP) was determined by staining with JC-1 probe and imaging using a fluorescence microscope at ×200 magnification. (B) MDBK cells were treated as described above for the indicated time periods. The percentage of green fluorescent cells (indicating reduced MMP) was quantified by flow cytometry. Values shown are the mean ± SD (n = 4). #*P*<0.05, ##*P*<0.01, and ###*P*<0.001 compared to the medium-treated control group; *P<0.05 and **P<0.01 compared to the cells exposed to Cd^2+^ alone (10 or 50 μM).

Mitochondria are both the source and the target of ROS [21], and we therefore explored the impact of Cd^2+^ exposure on mitochondrial MMP. MDBK cells were exposed to Cd^2+^ and/or Zn^2+^ for 6 h prior to MMP evaluation by JC-1 fluorescence imaging of (Fig. 3A). Exposure to 10 μM or 50 μM Cd^2+^ caused green JC-1 fluorescence in MDBK cells, indicating that the MMP was reduced and that the mitochondria were therefore depolarized. Addition of Zn^2+^ reversed this Cd^2+^-induced decline in MMP, with the best effect observed in the presence of 50 μM of Zn^2+^. Treatment with 10 μM or 50 μM Zn^2+^ alone did not obviously alter MMP in MDBK cells.

To examine the time-course of this Zn^2+^-mediated protection of MMP, the amounts of green fluorescence-emitting cells were quantified by flow cytometry over time (3–24 h, Fig. 3B). This showed that Cd^2+^ exposure led to dose- and time-dependent mitochondrial depolarization. At all of the examined time points, exposure to 50 μM Cd^2+^ resulted in around ten-fold more mitochondrial damage than did exposure to 10 μM Cd^2+^. Consistent with our previous observations, the protective effects of Zn^2+^ supplementation on MMP were conditional upon the Cd^2+^ concentration and the exposure time. Zn^2+^ afforded the most obvious protection against the lower Cd^2+^ concentration (10 μM) or at the early stages of exposure to the higher Cd^2+^ concentration (50 μM). Taken together, these results further illustrated that Zn^2+^ had the capacity to counteract Cd^2+^-induced toxicity in MDBK cells. However, exposure to high levels of Cd^2+^ could result in irreversible damage to bovine renal cells, which would not be alleviated by Zn^2+^ administration.

### Effect of Zn^2+^ on MTF-1 and MT expression in Cd^2+^-exposed MDBK cells

**Figure 4:**
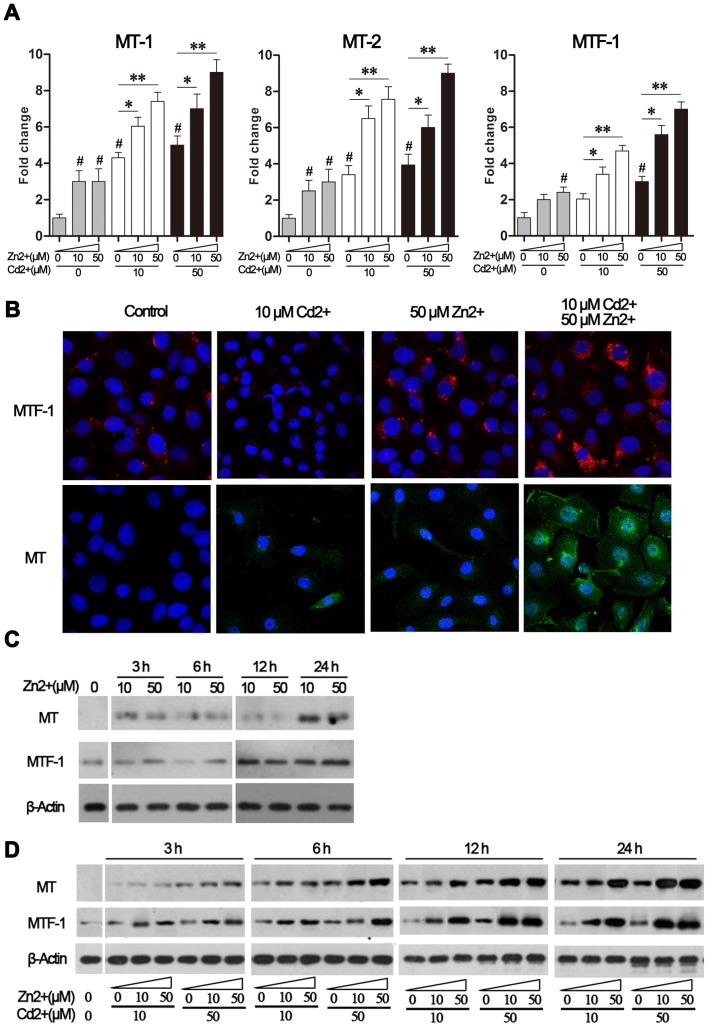
Zn^2+^ and Cd^2+^ increased expression of MTs and MTF-1. MDBK cells were treated with either Cd^2+^ alone (0, 10, 50 μM), or in combination with Zn^2+^ (0, 10, 50 μM), for up to 24 h. (A) MT-1, MT-2, and MTF-1 mRNA levels were determined by real-time PCR after 6-h treatments. Target gene threshold cycle (Ct) values were normalized to those of the reference gene (GAPDH) and the data were expressed as fold change over the medium-treated control sample, which was set as 1. The results are presented as the mean ± SD (n = 4). #*P*<0.05 compared to the medium-treated control group; *P<0.05, and **P<0.01 compared to the cells exposed to Cd^2+^ alone (10 or 50 μM). (B) Protein levels of MTs and MTF-1 after 6-h treatments were evaluated by fluorescence microscopy at ×400 magnification following staining with anti-MTF-1 or anti-MT antibodies. (C) Protein levels of MTs and MTF-1 in response to Zn^2+^ treatment (10 or 50 μM) at the indicated time points, as determined by western blot analysis. (D) Protein levels of MTs and MTF-1 in response to exposure to both Zn^2+^ and Cd^2+^ (10 and 50 μM, respectively) at the indicated time points, as determined by western blot analysis. In (C) and (D), expression of β-actin was used as the loading control.

Intracellular MTs are essential for Cd^2+^ detoxification, and the zinc finger transcription factor MTF-1 plays a critical role in metal-induced MT transcription. We therefore determined the impact of Cd^2+^ and/or Zn^2+^ exposure on MT-1, MT-2, and MTF-1 mRNA levels in MDBK cells using real-time PCR. As shown in Fig. 4A, 6-h exposure to Cd^2+^ or Zn^2+^ alone led to comparable up-regulation of MT-1 and MT-2 mRNA levels. Moreover, incubation with both Zn^2+^ and Cd^2+^ substantially enhanced transcription of these MTs. Co-treatment with both Zn^2+^ and Cd^2+^ significantly enhanced MTF-1 mRNA levels in MDBK cells (Fig. 4A). We also observed increased levels of MT and MTF-1 proteins in MDBK cells following 6-h exposure to Cd^2+^ and Zn^2+^ using fluorescence imaging (Fig. 4B).

To study the kinetics of Zn^2+^- and Cd^2+^-mediated up-regulation of MT-1, MT-2, and MTF-1, MDBK cells were exposed to the same conditions as those presented in Fig. 4A prior to quantification of MTs and MTF-1 protein levels by western immunoblotting at a range of time points. As shown in Fig. 4C, levels of the MTs and MTF-1 proteins peaked dose-independently after 24 h and 12 h Zn^2+^ treatment, respectively. The addition of Zn^2+^ to Cd^2+^-exposed cells enhanced the MTs and MTF-1 protein levels in a dose- and time-dependent manner (Fig. 4D). These findings were consistent with the observations presented in Fig. 4A and indicated that Cd^2+^ and Zn^2+^ stimulated expression of MTs and MTF-1 in a synergistic manner.

### Effects of Zn^2+^ administration on Cd^2+^ accumulation in MDBK cells

**Figure 5:**
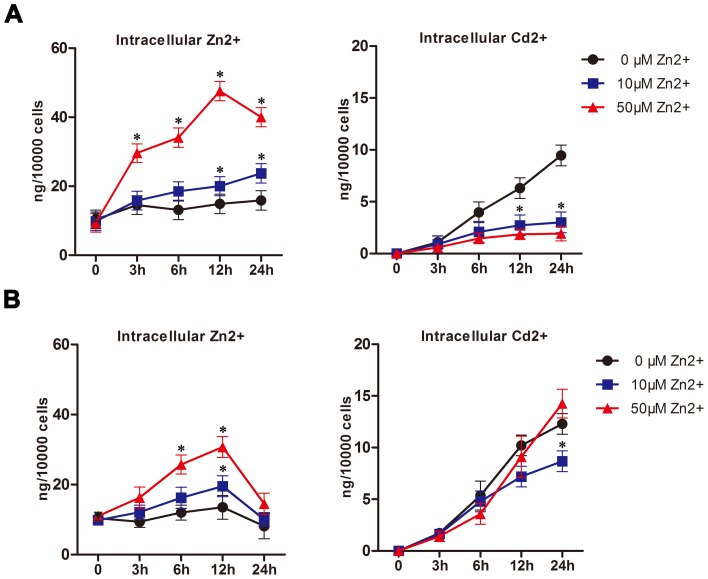
Quantification of intracellular Cd^2+^ and Zn^2+^. MDBK cells were treated with 10 μM (A) or 50 μM (B) Cd^2+^, alone or in combination with Zn^2+^ (0, 10, or 50 μM). At the indicated time points, the intracellular levels of Zn^2+^ and Cd^2+^ (ng/1×10^4^ cells) were determined by ICP-MS, as described in the Materials and Methods section. The results represent the mean ± SD (n = 4). *P<0.05, compared to the cells without Zn^2+^ supplementation.

As Zn^2+^ protected MDBK cells from Cd^2+^-induced cytotoxicity and up-regulated MT proteins, we next explored whether Zn^2+^ administration was able to reduce intracellular Cd^2+^ accumulation. MDBK cells were treated with 10 μM (Fig. 5A) or 50 μM (Fig. 5B) Cd^2+^ in the presence or absence of Zn^2+^ at indicated concentrations (0 to 50 μM), and the intracellular levels of Zn^2+^ and Cd^2+^ were determined over time using ICP-MS. In cells exposed to 10 μM Cd^2+^, addition of Zn^2+^ resulted in a dose-dependent reduction in the intracellular Cd^2+^ level, which was associated with increased levels of intracellular Zn^2+^ (Fig. 5A). In contrast, the inhibitory impact of Zn^2+^ supplementation on Cd^2+^ uptake was not obvious in cells exposed to a high concentration of Cd^2+^ (50 μM). Moreover, exposure to a high concentration of Zn^2+^ (50 μM) even tended to increase intracellular Cd^2+^ levels (Fig. 5B, right panel). Notably, the intracellular Zn^2+^ level under these circumstances was comparable to that of control samples after 24 h treatment, even though transient Zn^2+^ absorption was detected (Fig. 5B, left panel). These results indicated that Zn^2+^ supplementation was able to prevent Cd^2+^ uptake into MDBK cells, but its efficacy was dependent on the level of Cd^2+^ exposure.

## Discussion

This study demonstrated that Cd^2+^ exposure was cytotoxic to a bovine cell line (MDBK) and indicated that Zn^2+^ supplementation had protective effects against this Cd^2+^-mediated cytotoxicity. In line with previous observations in other species [23], [24], Cd^2+^ triggered MDBK apoptotic cell death in a time- and dose-dependent manner. This was associated with a Cd^2+^-induced increase in ROS accumulation in these cells, and mitochondrial depolarization. Addition of Zn^2+^ counteracted this Cd^2+^-induced oxidative stress and restored the MMP; effects which may explain the Zn^2+^-mediated protection against Cd^2+^-induced apoptosis death in these cells. Our findings also indicated that Zn^2+^ was less protective in cells exposed to the higher Cd^2+^ concentration used in this study (50 μM), especially when cells were treated with prolonged Cd^2+^ exposure (>12 h), indicating that early administration of Zn^2+^ is important in the prevention of Cd^2+^ toxicity.

Mitochondria are attacked by ROS, and ROS elimination may therefore protect mitochondria from structural damage and reduce the release of pro-apoptotic factors. Zn^2+^ plays an essential role in maintaining the wild-type conformation of copper-zinc superoxide dismutase 1 (SOD1), which plays a key role in the antioxidant defense system [25]. Moreover, SOD1 protects cells from apoptosis by inhibiting caspase-9 activation and release of mitochondrial cytochrome *c* [25], and by reducing chronic endoplasmic reticulum stress [26]. Another endogenous defense system against metal-induced oxidative stress involves glutathione (GSH) and GSH-associated enzymes [27]. GSH prevents Cd^2+^ intake through down-regulating expression of the Cd^2+^ transporter ZIP8 [28]. Effect of Zn on maintaining normal GSH levels has been investigated in several species such as human, mouse, fish and hamster [17], [29]–[31]. It would therefore be interesting to characterize the role of SOD1 and GSH-associated enzymes in Zn^2+^-mediated protection from Cd^2+^ cytotoxicity in future studies.

The conserved function of MTs in Cd^2+^ detoxification has been demonstrated in many species including yeast, zebra fish, mouse, and humans [7], [32]. However, large tissue- and individual-variation in the expression of MTs and their transcription regulatory factor, MTF-1, have been observed and these may affect the cellular response to Cd^2+^-induced toxicity [33]. In this study, we systematically assessed the regulation of MTs and MTF-1 upon exposure to Cd^2+^ and/or Zn^2+^ in MDBK cells. Our results showed that treatment with either Zn^2+^ or Cd^2+^ alone resulted in comparable up-regulation of MTs, indicating that they may be involved in MDBK cellular defense against toxic levels of heavy metals, consistent with previous reports in other species [10]. Notably, co-exposure to both Zn^2+^ and Cd^2+^ resulted in a remarkable dose- and time-dependent increase of MT-1 and MT-2 levels in MDBK cells. In line with previous *in vivo* and *in vitro* studies demonstrating that MTF-1 was the major regulator of MT gene expression, we also observed strong up-regulation of MTF-1 in MDBK cells exposed to Zn^2+^ and Cd^2+^. Our results indicated that the presence of Cd^2+^ promoted Zn^2+^-mediated up-regulation of MTF-1 and MTs. Cd^2+^ has a high binding affinity to MTs and may displace MT-bound Zn^2+^, triggering activation and nuclear translocation of MTF-1. Nevertheless, we cannot exclude the role of other factors affecting MT gene expression, such as activation of the antioxidant response element or epigenetic modification of the MT promoters [34].

Although co-exposure to Cd^2+^ and Zn^2+^ led to dose- and time-dependent up-regulation of MT protein levels, this did not protect MDBK cells from intracellular accumulation of Cd^2+^ or from apoptosis during exposure to a high Cd^2+^ concentration (50 μM), suggesting that MT-mediated sequestration may not be sufficient to prevent Cd^2+^ toxicity in these cells. However, we found that when cells were exposed to low levels of Cd^2+^ (10 μM), supplementation of Zn^2+^ could efficiently reduce intracellular Cd^2+^ content, probably through MT-mediated detoxification. Our finding that this was not observed when cells were exposed to high levels of Cd^2+^ (50 μM) was consistent with a previous report in a rat model [35]. Moreover, the intracellular level of Zn^2+^ even decreased to basal levels at 24 h after challenge, indicating that high levels of Cd^2+^ disturbed Zn^2+^ uptake in MDBK cells; this finding was also consistent with a previous report in a rat model [36]. These results also explained our findings that Zn^2+^ supplementation provided notable protection against Cd^2+^-triggered cytotoxicity at early time points or upon exposure to the lower concentration of Cd^2+^ tested (10 μM).

Collectively, the results from this study revealed that Zn^2+^ had protective effects against Cd^2+^-induced cytotoxicity in MDBK cells. We further demonstrated that Zn^2+^ administration efficiently reduced Cd^2+^ uptake in cells exposed to 10 μM Cd^2+^. These findings fill a current gap in our understanding of the role of Zn^2+^ supplementation in maintaining animal health; dietary Zn^2+^ may help to prevent Cd^2+^ accumulation and toxicity in cattle and other farm animals. Further *in vivo* investigations to evaluate the most effective dosage are warranted.

## Supporting Information

Table S1 **Real-time polymerase chain reaction primers.**
(DOCX)

## References

[1] Prozialeck WC, Edwards JR, Nebert DW, Woods JM, Barchowsky A, et al (2007) The Vascular System as a Target of Metal Toxicity. Toxicological Sciences 102: 207–218. 17947343 10.1093/toxsci/kfm263 PMC2752624

[2] Järup L, Åkesson A (2009) Toxicology and Applied Pharmacology. Toxicology and Applied Pharmacology 238: 201–208. 19409405 10.1016/j.taap.2009.04.020

[3] Bernhoft RA (2013) Cadmium Toxicity and Treatment. The Scientific World Journal 2013: 1–7. 10.1155/2013/394652 PMC3686085 23844395

[4] Bhattacharyya MH (2009) Cadmium osteotoxicity in experimental animals: Mechanisms and relationship to human exposures. Toxicology and Applied Pharmacology 238: 258–265. 19463839 10.1016/j.taap.2009.05.015 PMC2826165

[5] Peralta-Videa JR, Lopez ML, Narayan M, Saupe G, Gardea-Torresdey J (2009) The biochemistry of environmental heavy metal uptake by plants: Implications for the food chain. The International Journal of Biochemistry & Cell Biology 41: 1665–1677. 19433308 10.1016/j.biocel.2009.03.005

[6] Zasadowski A, Barski D, Markiewicz K, Zasadowski Z, Spodniewska A, et al (1999) Levels of Cadmium Contamination of Domestic Animals (Cattle) in the Region of Warmia and Masuria. Polish Journal of Environmental Studies 8 6: 443–446.

[7] Vašák M, Meloni G (2011) Chemistry and biology of mammalian metallothioneins. J Biol Inorg Chem 16: 1067–1078. 21647776 10.1007/s00775-011-0799-2

[8] Saydam N, Adams TK, Steiner F, Schaffner W, Freedman JH (2002) Regulation of metallothionein transcription by the metal-responsive transcription factor MTF-1: identification of signal transduction cascades that control metal-inducible transcription. J Biol Chem 277: 20438–20445. 11923282 10.1074/jbc.M110631200

[9] Duprez J, Roma LP, Close A-F, Jonas J-C (2012) Protective Antioxidant and Antiapoptotic Effects of ZnCl2 in Rat Pancreatic Islets Cultured in Low and High Glucose Concentrations. PLoS ONE 7: e46831. 23056475 10.1371/journal.pone.0046831 PMC3463538

[10] Klaassen CD, Liu J, Diwan BA (2009) Toxicology and Applied Pharmacology. Toxicology and Applied Pharmacology 238: 215–220. 19362100 10.1016/j.taap.2009.03.026 PMC2740813

[11] Thévenod F, Lee W-K (2013) Cadmium and cellular signaling cascades: interactions between cell death and survival pathways. Arch Toxicol 87: 1743–1786. 23982889 10.1007/s00204-013-1110-9

[12] Lee WK (2006) Caspase-dependent and -independent pathways for cadmium-induced apoptosis in cultured kidney proximal tubule cells. AJP: Renal Physiology 291: F823–F832. 16597613 10.1152/ajprenal.00276.2005

[13] Kowaltowski AJ, de Souza-Pinto NC, Castilho RF, Vercesi AE (2009) Mitochondria and reactive oxygen species. Free Radical Biology and Medicine 47: 333–343. 19427899 10.1016/j.freeradbiomed.2009.05.004

[14] Brzóska MM, Moniuszko-Jakoniuk J (2001) Interactions between cadmium and zinc in the organism. Food Chem Toxicol 39: 967–980. 11524135 10.1016/s0278-6915(01)00048-5

[15] Formigari A, Irato P, Santon A (2007) Zinc, antioxidant systems and metallothionein in metal mediated-apoptosis: Biochemical and cytochemical aspects. Comparative Biochemistry and Physiology Part C: Toxicology & Pharmacology 146: 443–459. 17716951 10.1016/j.cbpc.2007.07.010

[16] Bishop GM, Dringen R, Robinson SR (2007) Zinc stimulates the production of toxic reactive oxygen species (ROS) and inhibits glutathione reductase in astrocytes. Free Radical Biology and Medicine 42: 1222–1230. 17382203 10.1016/j.freeradbiomed.2007.01.022

[17] Tang W, Sadovic S, Shaikh ZA (1998) Nephrotoxicity of cadmium-metallothionein: protection by zinc and role of glutathione. Toxicology and Applied Pharmacology 151: 276–282. 9707504 10.1006/taap.1998.8465

[18] Belyaeva EA, Belyaeva EA, Belyaeva EA, Dymkowska D, Dymkowska D, et al (2008) Mitochondria as an important target in heavy metal toxicity in rat hepatoma AS-30D cells. 231: 34–42. 10.1016/j.taap.2008.03.017 18501399

[19] Aimola P, Carmignani M, Volpe AR, Di Benedetto A, Claudio L, et al (2012) Cadmium Induces p53-Dependent Apoptosis in Human Prostate Epithelial Cells. PLoS ONE 7: e33647. 22448262 10.1371/journal.pone.0033647 PMC3308998

[20] Kondoh M, Araragi S, Sato K, Higashimoto M, Takiguchi M, et al (2002) Cadmium induces apoptosis partly via caspase-9 activation in HL-60 cells. Toxicology 170: 111–117. 11750088 10.1016/s0300-483x(01)00536-4

[21] Herrera B, Alvarez AM, Sánchez A, Fernández M, Roncero C, et al (2001) Reactive oxygen species (ROS) mediates the mitochondrial-dependent apoptosis induced by transforming growth factor (beta) in fetal hepatocytes. FASEB J 15: 741–751. 11259392 10.1096/fj.00-0267com

[22] Shen C, James SA, de Jonge MD, Turney TW, Wright PFA, et al (2013) Relating Cytotoxicity, Zinc Ions, and Reactive Oxygen in ZnO Nanoparticle-Exposed Human Immune Cells. Toxicological Sciences 136: 120–130. 23997113 10.1093/toxsci/kft187

[23] Kim J, Sharma RP (2006) Cadmium-induced Apoptosis in Murine Macrophages is Antagonized by Antioxidants and Caspase Inhibitors. Journal of Toxicology and Environmental Health, Part A 69: 1181–1201. 16728380 10.1080/15287390600631144

[24] Xu S, Pi H, Chen Y, Zhang N, Guo P, et al (2013) Cadmium induced Drp1-dependent mitochondrial fragmentation by disturbing calcium homeostasis in its hepatotoxicity. Cell Death and Disease 4: e540–10. 23492771 10.1038/cddis.2013.7 PMC3615741

[25] Sugawara T (2002) Overexpression of SOD1 protects vulnerable motor neurons after spinal cord injury by attenuating mitochondrial cytochrome c release. The FASEB Journal Dec 16 14: 1997–9. 10.1096/fj.02-0251fje 12368231

[26] Homma K, Fujisawa T, Tsuburaya N, Yamaguchi N, Kadowaki H, et al (2013) SOD1 as a Molecular Switch for Initiating the Homeostatic ER Stress Response under Zinc Deficiency. Molecular Cell 52: 75–86. 24076220 10.1016/j.molcel.2013.08.038

[27] Jozefczak M, Remans T, Vangronsveld J, Cuypers A (2012) Glutathione Is a Key Player in Metal-Induced Oxidative Stress Defenses. IJMS 13: 3145–3175. 22489146 10.3390/ijms13033145 PMC3317707

[28] Aiba I, Hossain A, Kuo MT (2008) Elevated GSH Level Increases Cadmium Resistance through Down-Regulation of Sp1-Dependent Expression of the Cadmium Transporter ZIP8. Molecular Pharmacology 74: 823–833. 18556457 10.1124/mol.108.046862 PMC2574563

[29] Lange A, Ausseil O, Segner H (2002) Alterations of tissue glutathione levels and metallothionein mRNA in rainbow trout during single and combined exposure to cadmium and zinc. Comp Biochem Physiol C Toxicol Pharmacol 131: 231–243. 11912048 10.1016/s1532-0456(02)00010-8

[30] Omata Y, Salvador GA, Supasai S, Keenan AH, Oteiza PI (2013) Decreased Zinc Availability Affects Glutathione Metabolism in Neuronal Cells and in the Developing Brain. Toxicological Sciences 133: 90–100. 23377617 10.1093/toxsci/kft022 PMC3627551

[31] Seagrave J, Tobey RA, Hildebrand CE (1983) Zinc effects on glutathione metabolism relationship to zinc-induced protection from alkylating agents. Biochemical Pharmacology 32: 3017–3021. 6639670 10.1016/0006-2952(83)90243-5

[32] Cheukb WK, Chana PC-Y, Chan KM (2008) Cytotoxicities and induction of metallothionein (MT) and metal regulatory element (MRE)-binding transcription factor-1 (MTF-1) messenger RNA levels in the zebrafish (Danio rerio) ZFL and SJD cell lines after exposure to various metal ions. Aquatic Toxicology 89: 103–112. 18639347 10.1016/j.aquatox.2008.06.006

[33] Lindert U, Cramer M, Meuli M, Georgiev O, Schaffner W (2009) Metal-responsive transcription factor 1 (MTF-1) activity is regulated by a nonconventional nuclear localization signal and a metal-responsive transactivation domain. Mol Cell Biol 29: 6283–6293. 19797083 10.1128/MCB.00847-09 PMC2786702

[34] Campagne MV, Thibodeaux H, van Bruggen N, Cairns B, Lowe DG (2000) Increased binding activity at an antioxidant-responsive element in the metallothionein-1 promoter and rapid induction of metallothionein-1 and -2 in response to cerebral ischemia and reperfusion. J Neurosci 20: 5200–5207. 10884303 10.1523/JNEUROSCI.20-14-05200.2000 PMC6772313

[35] Rogalska J, Pilat-Marcinkiewicz B, Brzóska MM (2011) Chemico-Biological Interactions. Chemico-Biological Interactions 193: 191–203. 21627960 10.1016/j.cbi.2011.05.008

[36] Kaur J, Sharma N, Attri S, Gogia L, Prasad R (2006) Kinetic characterization of Zinc transport process and its inhibition by Cadmium in isolated rat renal basolateral membrane vesicles: In vitro and In vivo studies. Mol Cell Biochem 283: 169–179. 16444600 10.1007/s11010-006-2676-9

