# Supplementary material for: Zinc Supplementation Protects against Cadmium Accumulation and Cytotoxicity in Madin-Darby Bovine Kidney Cells
Source: PLoS One. 2014 Aug 8;9(8):e103427. doi: 10.1371/journal.pone.0103427 (PMC4126686; doi:10.1371/journal.pone.0103427)
Supplement: Table S1 — Real-time polymerase chain reaction primers. (DOCX) [file pone.0103427.s001.docx]

| Gene | P Primer sequence（5′→3′） |
| --- | --- |
| *mt-1* | Forward: GCGTCACCACGACTTCAAC  Reverse: GTCACATCAGGCACAGCAC |
| *mt-2* | Forward: AACTGCCGCCTCCATTCGC  Reverse: AGCACTTCGCACAGCCCAC |
| *mtf-1* | Forward: CTGCTCCCTCCCTAGGAACT  Reverse: TGGAGGTGCGGAAGAAACTC |
| *gapdh* | Forward: CCTTCATTGACCTTCACTACATGGTCTA  Reverse: TGGAAGATGGTGATGGCCTTTCCATTG |
